# Elderly Woman With No Autoimmune Disease With Aseptic Meningitis Caused by Celecoxib

**DOI:** 10.7759/cureus.55348

**Published:** 2024-03-01

**Authors:** Masaki Takigawa, Hiroyuki Tanaka, Takashi Kobayashi, Yuto Katahara, Masako Kinoshita, Masayuki Masuda, Rika Iwakiri

**Affiliations:** 1 Department of Clinical Pharmaceutics, Faculty of Pharmaceutical Sciences, Toho University, Chiba, JPN; 2 Department of Practical Pharmacy, Faculty of Pharmaceutical Sciences, Toho University, Chiba, JPN; 3 Department of General Internal Medicine, Daisan Hospital, Jikei University, School of Medicine, Tokyo, JPN; 4 Department of Pharmacy, Tokyo Metropolitan Institute for Geriatrics and Gerontology, Tokyo, JPN; 5 Department of Geriatrics, Tokyo Metropolitan Institute for Geriatrics and Gerontology, Tokyo, JPN

**Keywords:** cyclooxygenase-2 selective agents, nonsteroidal anti-inflammatory drug, japanese adverse drug event report database, disproportionality analysis, aseptic meningitis

## Abstract

Nonsteroidal anti-inflammatory drug (NSAID)-induced aseptic meningitis (NIAM) is frequently reported in patients with autoimmune disease. Ibuprofen-induced NIAM is the most common case report of NIAM. We report a patient without autoimmune disease who developed NIAM following oral celecoxib administration. A literature review and survey of cases registered in the Japanese Adverse Drug Event Report (JADER) database is also provided. A 73-year-old woman with no autoimmune disease developed a headache the day after taking celecoxib, and NIAM was suspected. The headache resolved quickly following celecoxib discontinuation. Although lumbar puncture was not available in this case, bacterial or viral meningitis was negative, and NIAM could not be ruled out. This case involved an older adult patient without an autoimmune disease, with celecoxib as the causative NSAID. A literature review found numerous cases of autoimmune diseases in younger patients. To date, only one case of celecoxib-induced NIAM has been reported. Analysis of NIAM cases in JADER revealed an onset time of approximately three days. JADER analysis indicated that NIAM tended to occur immediately after administration, although the onset with cyclooxygenase-2 selective agents might be slower.

## Introduction

Aseptic meningitis is a meningitis type where no pathogenic microorganisms are found in cerebrospinal fluid (CSF). The reported causes of this condition include systemic diseases affecting the meninges, neoplastic or paraneoplastic meningitis, viruses, and drugs [1]. Immunoglobulin preparations, antimicrobial agents, vaccines, and nonsteroidal anti-inflammatory drugs (NSAIDs), commonly used in clinical practice, are some of the agents that reportedly induce aseptic meningitis [2,3]. NSAID-induced aseptic meningitis caused by NSAIDs is frequently reported [4]. The causative agents of NSAID-induced aseptic meningitis (NIAM) include ibuprofen, sulindac, naproxen, diclofenac, rofecoxib, and celecoxib, with ibuprofen being the most frequently reported [5]. The first documented case of drug-induced aseptic meningitis was linked to ibuprofen [6], and ibuprofen-induced meningitis has been reported in recent years [7-9]. The pathogenesis of NIAM suggests that proteins in the CSF bind to hapten drugs as carriers, forming complex antigens that induce meningitis through a type III allergic mechanism [10]. NIAM generally resolves quickly with the discontinuation of NSAIDs. CSF findings are variable and nonspecific. NIAM is more prevalent in patients with autoimmune diseases such as systemic lupus erythematosus, Sjögren’s syndrome, and mixed connective tissue disease [3,11]. Here, we present a case of NIAM in a patient without autoimmune disease following oral celecoxib administration. Additionally, we conducted a literature review and analyzed cases from the Japanese Adverse Drug Event Report (JADER) database.

## Case presentation

A 73-year-old woman independently performed activities of daily living (ADL). Five days before admission, the patient fell into the bathroom. Four days before admission, the patient had experienced pain in the thighs, lower legs, and buttocks. Two days before admission, her buttock pain worsened, and she experienced numbness at night. Subsequently, she had difficulty rising and sitting down and called for emergency medical assistance. Three days before admission, the patient exhibited decreased urine output, followed by a left occipital headache the day before admission. Table 1 outlines the laboratory data upon admission.

**Table 1:** Laboratory data on admission

Characteristics	Values	Reference range
White blood cell	9,490 /µL	3,500-8,500 /µL
Neutrophils	76.5 %	38-57 %
Hemoglobin	14.3 g/dL	11.5-15.0 g/dL
Platelet	256×10³/µL	150-350 ×10³/µL
Total protein	7.0 g/dL	6.7-8.2 g/dL
Albumin	3.8 g/dL	3.9-5.2 g/dL
Aspartate aminotransferase	30 U/L	10-35 U/L
Alanine aminotransferase	34 U/L	5-40 U/L
Serum creatinine	0.81 mg/dL	0.4-0.8 mg/dL
Blood urea nitrogen	20 mg/dL	8-20 mg/dL
Sodium	130 mEq/L	136-145 mEq/L
Potassium	3.7 mEq/L	3.6-4.8 mEq/L

**Figure 1:**
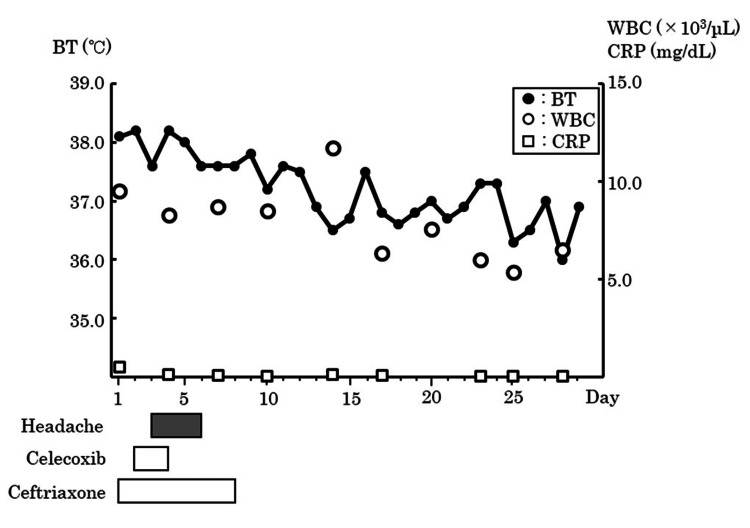
Clinical course of the patient BT, body temperature; WBC, white blood cell; CRP, C-reactive protein

Following admission, she developed a fever of 38.1°C. Due to suspicion of urinary tract infection secondary to urinary retention, 2 g ceftriaxone was initiated after obtaining blood and urine cultures (blood pressure: 157/87 mmHg; heart rate: 103 bpm; temperature: 38.1 °C at admission). Loxoprofen sodium tape was started on Day 1 of admission for leg pain, and celecoxib tablets 200 mg/2× (200 mg/dose only for the first time) on Day 2. On Day 3, the patient complained of a headache, which worsened on Day 4 with no improvement, and the fever persisted. The patient did not present symptoms of meningeal irritation. At this juncture, the potential for aseptic meningitis induced by celecoxib could not be dismissed; consequently, celecoxib was halted. Following celecoxib discontinuation, the headache improved by Day 7. Ceftriaxone, prescribed for urinary tract infection treatment, was ceased on Day 8 due to concerns of ceftriaxone encephalopathy and drug fever. Bacterial meningitis was ruled out in this case due to the absence of jolt accentuation, minimal inflammation, and negative blood culture results. The patient was referred to a neurologist, who stated that the possibility of bacterial meningitis was extremely low. As the headache improved despite ceftriaxone administration, ceftriaxone encephalopathy was ruled out. On Day 10, we conducted antibody tests for Epstein-Barr, varicella-zoster, herpes simplex, and mumps viruses to explore the etiology of aseptic meningitis, revealing pre-existing infection in the patient. Furthermore, the vitamin B1, vitamin B12, folic acid, and T-SPOT levels were normal. The patient was believed to have headaches caused by NSAIDs associated with autoimmune diseases. However, tests for rheumatoid arthritis, systemic lupus erythematosus, Sjögren’s syndrome, and mixed connective tissue disease were negative. As a lumbar puncture was not performed, a conclusive diagnosis could not be established. Nevertheless, the possibility of NSAID-induced aseptic meningitis could not be disregarded. The patient’s body temperature, white blood cell count, and C-reactive protein level during hospitalization are shown in Figure 1.

## Discussion

The current case exhibited two primary features. First, celecoxib was identified as the causative NSAID. Second, the patient was an older adult and lacked an autoimmune disease. Celecoxib was the causative NSAID used in this patient with NIAM. A literature review was performed using the Igaku Chuo Zasshi and PubMed databases, gathering Japanese case reports in both Japanese and English from January 2000 to March 2023, utilizing the keywords ‘nonsteroidal anti-inflammatory drugs’ and ‘aseptic meningitis’ [12-20]. Table 2 summarizes the characteristics of patients with NIAM (sex, age, causative NSAIDs, presence of autoimmune disease, time-to-NIAM onset, and outcomes) as reported previously.

**Table 2:** Case at Tokyo Metropolitan Institute for Geriatrics and Gerontology and review of the literature MCTD, mixed connective tissue disease; RNP, ribonucleoprotein; SLE, systemic lupus erythematosus; UCTD, undifferentiated connective tissue disease; ‐, Information could not be verified from literature

Patient No.	Author	Sex	Age	Causative NSAIDs	Autoimmune disease	Onset interval (day)	Outcome
1	Our case	Female	73	Celecoxib	None	1	Recovery
2	Mimori et al (2000) [12]	Female	23	Ibuprofen	MCTD	‐	Recovery
3	Mimori et al (2000) [12]	Male	48	Ibuprofen	MCTD	‐	Recovery
4	Mimori et al (2000) [12]	Female	22	Ibuprofen	SLE	‐	Recovery
5	Kanda et al (2003) [13]	Female	25	Sulindac	UCTD	2	Recovery
6	Okada et al (2003) [14]	Female	38	Loxoprofen	UCTD	1	Recovery
7	Okada et al (2003) [14]	Male	44	Sulindac	SLE	1	Recovery
8	Okada et al (2003) [14]	Female	19	Etodolac	SLE	3	Recovery
9	Okada et al (2003) [14]	Female	39	Ibuprofen	MCTD	3	Recovery
10	Okada et al (2003) [14]	Female	28	Loxoprofen	MCTD	2	Recovery
11	Sekiguchi et al (2003) [15]	Female	35	Zaltoprofen	Sjogren syndrome	1	Recovery
12	Takashima et al (2004) [16]	Male	34	Diclofenac	None	2	Recovery
13	Muneyuki et al (2010) [17]	Female	46	Diclofenac	SLE	‐	Recovery
14	Asano et al (2017) [18]	Female	26	Loxoprofen, Diclofenac	SLE	‐	Recovery
15	Hamada et al (2018) [19]	Female	72	Acetaminophen, Celecoxib	MCTD	‐	Recovery
16	Matsui et al (2018) [20]	Female	19	Loxoprofen	Raynaud phenomenon, anti-RNP antibody-positive	1	Recovery

In a Japanese case report, celecoxib was identified as the causative NSAID in only one instance (patient number 15) [19]. Outside Japan, there was a single case report of NIAM where celecoxib was the causative NSAID [21]. Additionally, NIAM cases recorded in JADER, a Japanese database of adverse drug reactions, were examined. Table 3 presents the characteristics of these cases. Among the registered NIAM cases, 71 were attributed to loxoprofen, the most prevalent drug, followed by diclofenac (25 cases). Conversely, celecoxib was implicated in 11 cases, a lower frequency compared to the other two drugs. Hence, reports of NIAM induced by celecoxib are relatively infrequent.

**Table 3:** Baseline characteristics and clinical outcomes of patients extracted from the JADER database. NSAIDs, nonsteroidal anti-inflammatory drugs; NIAM, NSAID-induced aseptic meningitis; JADER, Japanese Adverse Drug Event Report

Characteristics	No. of patients (%)
Sex	
Male	57 (44.9)
Female	70 (55.1)
Age	
< 10	3 (2.4)
10-19	26 (20.5)
20-29	20 (15.7)
30-39	33 (26.0)
40-49	19 (15.0)
50-59	11 (8.7)
60-69	8 (6.3)
70-79	4 (3.1)
Unknown	3 (2.4)
NSAIDs and acetaminophen	
Acetaminophen	5 (3.9)
Aspirin	2 (1.6)
Celecoxib	11 (8.7)
Diclofenac	25 (19.7)
Etodolac	1 (0.8)
Ibuprofen	4 (3.1)
Indomethacin	1 (0.8)
Flurbiprofen	2 (1.6)
Lornoxicam	1 (0.8)
Loxoprofen	71 (55.9)
Meloxicam	1 (0.8)
Naproxen	2 (1.6)
Sulindac	1 (0.8)
Clinical outcome of NIAM	
Recovery	54 (42.5)
Remission	61 (48.0)
Sequelae	2 (1.6)
Unknown	10 (7.9)

**Figure 2:**
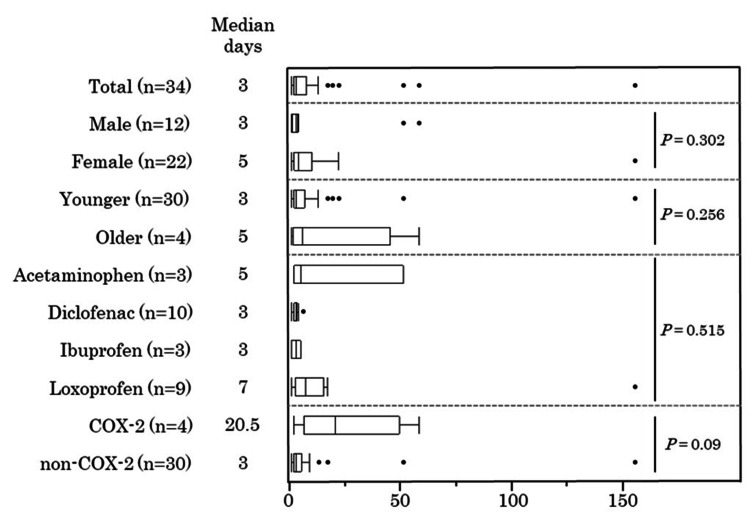
Comparison of time-to-onset among groups of patients with NSAID-induced aseptic meningitis enrolled in JADER. The Mann–Whitney U-test was used for the comparison of two groups, and a one-way analysis of variance followed by post-hoc Bonferroni correction was used for the comparison of multiple groups. Since the JADER database categorizes age in 10-year intervals, “younger” and “older” groups were classified to encompass individuals in their 50s or below and those in their 60s or above, respectively. The COX-2 selective agents are celecoxib, etodolac, and meloxicam. COX-2, cyclooxygenase-2; NSAIDs, nonsteroidal anti-inflammatory drugs; ADER, Japanese Adverse Drug Event Report This figure was created by the author.

Furthermore, in the present case, NIAM onset occurred within one day. Typically, drug-induced aseptic meningitis manifests within three days, although onset times vary widely [4]. A literature review also revealed that the onset of NIAM typically occurs within one to three days, consistent with the timing observed in our case. Furthermore, 34 NIAM cases registered in JADER were selected, excluding those without data on sex, age, clinical outcome, date of NSAID administration, date of NSAID initiation, date of NIAM onset, and number of days to NIAM onset based on patient background (Figure 2).

In the 34 NIAM cases extracted from the JADER database, the median onset of NIAM was three days. Similarly, in our case and the literature review, onset within three days was common. Therefore, caution should be exercised during the initial stages of NSAID use. Conversely, the median time to onset of NIAM with cyclooxygenase-2 (COX-2) selective agents alone was 20.5 days, surpassing that of non-COX-2 selective agents. In a case report of celecoxib-induced NIAM outside Japan, the onset of the condition was noted five days after initiation of treatment [21]. Highlighting case reports of rofecoxib-induced aseptic meningitis, a COX-2 selective agent akin to celecoxib, disease onset ranged from as short as three to five days in certain instances to as long as 7, 12, and 23 days following therapy initiation in others [22,23]. NIAM may develop slowly owing to the COX-2 selectivity of NSAID, which requires further study.

This case involves a 73-year-old woman without autoimmune diseases, whereas NIAM typically affects younger women. Most cases in the literature review were in younger age groups. Patient 15 was an exception, a 72-year-old woman with an autoimmune disease who developed NIAM on celecoxib [19]. The case shared similarities with ours regarding the older age of the patient but differed in the presence of autoimmune disease. In case reports of NIAM with celecoxib outside Japan [21], the patient was a 65-year-old woman without autoimmune disease, aligning with our case. These findings indicate that older adult patients might develop aseptic meningitis after celecoxib treatment, even without autoimmune disease. Given celecoxib’s demonstrated penetration into the central nervous system in animal studies [24], aseptic meningitis may be more prone to develop even in the absence of autoimmune disease.

The present case study has several limitations. First, lumbar puncture was not performed, and NIAM was not verified. However, we considered this patient as a possible NIAM for the following reasons: 1) Headache occurred from the start of NSAIDs, and the headache improved when the NSAIDs were discontinued; 2) Other possible causes of meningitis were ruled out; 3) The neurologist ruled out bacterial meningitis. Second, the quality of the data registered in the JADER database has not been verified and lacks the necessary case details, making under-reporting and reporting bias undeniable. Third, the number of case series from the reviewed literature and the JADER database was small. In particular, our discussion of the pathogenesis of NIAM following the administering of COX-2 selective agents of COX-2 selective agents is based on a small number of cases. Therefore, future studies should consider more cases and include cases from databases other than the JADER database. Increasing the number of cases would enable analyses that include concomitant medications and diseases.

## Conclusions

The present case of NIAM with celecoxib is characterized by an older adult patient with no autoimmune disease. Notably, NIAM can manifest in older adults and without autoimmune diseases. In the present case, the onset time was typical. Upon analyzing cases recorded in JADER, NIAM onset appeared to occur more gradually with COX-2 selective agents; however, further investigation with larger datasets is warranted. Despite reports of NSAIDs inducing aseptic meningitis, few studies have scrutinized patient populations. The accumulation of cases, including the present instance, is necessary to deepen our comprehension of NIAM. The current case, literature review, and our examination of JADER cases could contribute to advancing our understanding of NIAM.
